# PM_10_ Disrupts Mitochondrial Homeostasis in Corneal Epithelial Cells: Protective Effects of SKQ1

**DOI:** 10.3390/antiox15030284

**Published:** 2026-02-25

**Authors:** Mallika Somayajulu, Robert Wright, Farooq S. Muhammed, Sharon A. McClellan, Ahmed S. Ibrahim, Linda D. Hazlett

**Affiliations:** 1 Department of Ophthalmology, Visual and Anatomical Sciences, School of Medicine, Wayne State University, Detroit, MI 48201, USAahmed.ibrahim@wayne.edu (A.S.I.);; 2 Department of Pharmacology, School of Medicine, Wayne State University, Detroit, MI 48201, USA

**Keywords:** PM_10_, corneal epithelium, mitochondria, SKQ1

## Abstract

Airborne particulate matter with a diameter of <10 μm (PM_10_) can damage the corneal epithelium by inducing oxidative stress, disrupting the NRF2 antioxidant pathway, and triggering epithelial barrier dysfunction and inflammation. However, the role of mitochondria in mediating PM_10_-induced damage remains unexplored. This study investigated the impact of PM_10_ on mitochondrial homeostasis in both immortalized human corneal epithelial cells (HCE-2) and the mouse corneal epithelium, as well as the protective effects of SKQ1. For in vivo assessment, female C57BL/6 mice were exposed to either control air or PM_10_ (±SKQ1) in a whole-body exposure chamber for 2 weeks (3 h/day, 5 days/week, with weekends off). In vitro, HCE-2 cells were exposed to 100 μg/mL PM_10_ (±SKQ1) for 24 h, and mitochondrial function and morphology were evaluated. In vitro, PM_10_ significantly impaired mitochondrial function by reducing basal, maximal, and ATP-linked respiration; reserve capacity; and coupling efficiency compared to the control and SKQ1 groups. PM_10_ also downregulated mitofusin1 (MFN1) and optic atrophy1 (OPA1) and upregulated dynamin-related protein1 (DRP1) and mitochondrial fission protein1 (FIS1) in HCE-2 cells. In addition, PM_10_ exposure significantly decreased the mitochondrial membrane potential; mitochondrial DNA copy number; and cytochrome c oxidase subunit 4 isoform 1 (COX4i1), mitochondrial transcription factor A (TFAM), and peroxisome proliferator-activated receptor gamma coactivator 1 alpha (PGC-1α) levels. SKQ1 pre-treatment significantly attenuated these effects. In vivo, PM_10_ exposure significantly decreased the levels of MFN1, TFAM, COX4i1, and superoxide dismutase (SOD2), whereas SKQ1 treatment significantly reversed these effects. Overall, these findings demonstrate that PM_10_ exposure induces mitochondrial fragmentation, disrupts mitochondrial biogenesis and quality control, and reduces mitochondrial respiration, resulting in mitochondrial dysfunction. SKQ1 effectively reversed these changes, suggesting its potential as a therapeutic strategy to protect corneal epithelial cells from PM_10_-induced mitochondrial damage.

## 1. Introduction

Exposure to particulate matter less than 10 µm in aerodynamic diameter (PM_10_) is associated with increased morbidity and mortality [1,2,3]. Among its many adverse effects, PM_10_ exposure has been linked to significant ocular damage, including increased outpatient visits for eye discomfort [4], worsened tear film stability in patients with dry eye disease [5], and an elevated risk of Sjögren’s syndrome [6] and keratitis [7]. Our recent work has shown that PM_10_ exposure reduces the level of nuclear erythroid 2-related factor 2 (NRF2) and its transcriptional activity, leading to impaired antioxidant defenses and inflammation both in vitro [8,9] and in vivo [8]. NRF2 is a key regulator of antioxidant defenses [10], activated in response to oxidative stress, and its regulation occurs at multiple levels, including the transcriptional [11], post-transcriptional, translational [12], and protein stability levels [13]. Under basal conditions, NRF2 is bound to Kelch-like ECH-associated protein 1 (Keap1), a substrate adaptor of the Cullin-3-based E3 ubiquitin ligase in the cytosol, and remains inactive [10,14]. Upon activation, it translocates to the nucleus, where it binds to specific sites called antioxidant response elements (AREs) on DNA to regulate the expression of cell-protective genes [10,14]. NRF2 is a key regulator of mitochondrial structure, function, metabolism, and bioenergetics through its control of redox balance, antioxidant defense, and quality control pathways [15]. Mitochondria are key organelles essential for multiple facets of cellular function, including apoptosis [16] and energy production [16]. ATP production by mitochondria via oxidative phosphorylation (OxPhos) is the most efficient cellular pathway for generating energy [17]. During OxPhos, electrons are transported via the electron transport chain (ETC), creating a proton gradient that powers ATP synthase to generate ATP [17,18]. NRF2 reduces mitochondrial ROS by inducing genes involved in glutathione and NADPH production, thereby preserving ETC integrity and ATP production [15,19]. Structurally, NRF2 supports mitochondrial dynamics, regulates the balance between fission and fusion, and promotes mitophagy to remove damaged organelles [15]. It also influences biogenesis via crosstalk with transcription factors, such as PGC-1α and NRF1, ensuring adequate mitochondrial mass in response to stress [20,21]. Additionally, NRF2 stabilizes the mitochondrial membrane potential and prevents the opening of the permeability transition pore, thereby reducing susceptibility to calcium overload and apoptosis [15]. The cryoprotective role of NRF2 makes it an ideal candidate for therapeutic targeting. Therefore, compounds that activate NRF2 can be used as a therapeutic strategy to improve overall mitochondrial health and function [22]. One such compound is SKQ1 (10-(6-plastoquinonyl) decyltriphenylphosphonium), a mitochondrial antioxidant that accumulates in the inner mitochondrial membrane [23,24,25]. The administration of SKQ1 significantly increased the mRNA levels of NRF2 and NRF2-controlled genes encoding antioxidant enzymes in a rat model of hyperoxia [26]. Recently, we verified that SKQ1 is protective and restores NRF2 levels in primary human corneal epithelial cells (HCECs) and transformed human corneal epithelial cells (HCE-2) exposed to PM_10_ [8,9], as well as in the mouse cornea exposed to PM_10_ using a whole-body exposure chamber [8]. SKQ1 is also protective against oxidative stress-mediated damage in various animal models of disease [27,28,29,30]. Clinically, an ophthalmic formulation of SKQ1 (Visomitin) has been effective in preventing the pathology of anesthetic-induced dry eye syndrome after surgery or prolonged general anesthesia, demonstrating human relevance [31,32].

Therefore, our current study aimed to better understand the effects of PM_10_ on mitochondrial health and function in vitro in HCE-2 cells and in vivo in the mouse cornea and whether SKQ1 is protective.

## 2. Materials and Methods

### 2.1. Mice

Eight-week-old female C57BL/6 mice were purchased from the Jackson Laboratory (Bar Harbor, ME, USA). They were housed in compliance with the National Institutes of Health guidelines and treated humanely and in accordance with both the ARVO Statement for the Use of Animals in Ophthalmic and Vision Research) and the Institutional Animal Care and Use Committee of Wayne State University (IACUC 24-03-6665). The animal study protocol was approved by the Institutional Review Board (IACUC) of Wayne State University (IACUC 24-03-6665 and date of approval 30 May 2024–29 May 2027.

### 2.2. Whole-Body Exposure to PM_10_

For PM_10_ exposure, a whole-body exposure chamber (CH Technologies, Westwood, NJ, USA) was used, as previously described [8,33]. Briefly, mice were either exposed to control air or 0.5–1 mg/m^3^ PM_10_, for 3 h a day for 5 days per week for 2 weeks [33]. This range was chosen to reflect exposure levels similar to those reported in countries with high pollution levels [34,35,36].

### 2.3. SKQ1 Treatment

SKQ1 (catalog# 934826-68-3, BOC Sciences, Shirley, NY, USA) was used at a previously published dose of 7.5 µM to treat mice exposed to control air and PM_10_ [8,37]. Eyes were topically treated with 5 µL SKQ1 (7.5 µM) or PBS, three times on the day before the first chamber exposure and then once each day before exposure for two weeks, as previously described [8].

### 2.4. Separating Corneal Epithelium from Stroma

The corneal epithelium was separated from the stroma by modifying a previously published protocol [38]. Briefly, the corneas (right and left) of each mouse were dissected and incubated in 0.02 M EDTA-PBS buffer (pH 7.2) at 37 °C for 1 h, and the epithelium was separated from the stroma. For all in vivo experiments, four groups were tested: control air + PBS (control), control air + SKQ1 (SKQ1), PM_10_ + PBS (PM_10_), and PM_10_ + SKQ1.

### 2.5. Tissue Culture and Treatments

HCE-2 ([50.B1], catalog# CRL-11135, ATCC, Gaithersburg, MA, USA) cells were grown and cultured as previously reported [9]. PM_10_ (SRM 2787) was purchased from the National Institute of Standards and Technology (NIST, Gaithersburg, MD, USA). PM_10_ was resuspended in sterile phosphate-buffered saline (PBS) to prepare a 10 mg/mL stock solution and subsequently diluted to a final concentration of 100 µg/mL using culture medium. The PM_10_ dosage was chosen after performing a dose curve [9,39] and was used in all our previous studies [8,9,39,40]. More details regarding PM_10_ can be found online at https://tsapps.nist.gov/srmext/certificates/2787.pdf (accessed on 31 October 2025).

SKQ1 (catalog# 934826-68-3, BOC Sciences, Shirley, NY, USA) was dissolved in 50% ethanol to obtain a 162 mM stock solution, which was subsequently diluted to 50 nM using culture medium. The dosage of SKQ1 used in this study was chosen based on a dose curve and a previously published report [29] and was successfully used in our previous publications [8,9,39,40]. To test the protective effects of SKQ1, cells were pre-treated with 50 nM SKQ1 for 1 h and then exposed to 100 µg/mL PM_10_, as described previously [9]. An SKQ1 control was included, in which cells were treated with only 50 nM SKQ1 for 24 h. Untreated cells were used as the control. For all experiments, the cells were divided into four groups: control (untreated), SKQ1 (SKQ1-treated), PM_10_, and PM_10_ + SKQ1.

### 2.6. Quantitative Real-Time Polymerase Chain Reaction (qRT-PCR)

RNA extraction from HCE-2 cells (*n* = 3/group) and the mouse corneal epithelium (*n* = 5 per group) was performed using a previously described protocol [9]. Briefly, cDNA templates were generated from all samples. They were then diluted 20-fold with DEPC-treated water. SYBR Green PCR Master Mix (Bio-Rad Laboratories, Hercules, CA, USA) was used for qRT-PCR. For HCE-2 cells, the mRNA levels of *MFN-1*, *MFN2*; *OPA1*; *FIS1*; *DRP1*; *PGC-1α*; *TFAM*; and *COX4i1* were tested using real-time RT-PCR (CFX Connect real-time PCR detection system (Bio-Rad Laboratories, Hercules, CA). In the mouse corneal epithelium, the levels of *NRF2*, *SOD2*, *MFN1*, *MFN2*, *DRP1*, *PGC-1α*, *TFAM*, and *COX4i1* were analyzed. The primer sequences are listed in Table 1. The fold differences in gene expression were calculated relative to *18S rRNA* (in vitro) and β-actin (in vivo) using 2^−ΔΔCT^ and expressed as relative mRNA concentration ± SEM.

### 2.7. Protein Analysis

The Protein Simple Abby system (Biotechne, Minneapolis, MN, USA) was used to detect the levels of SOD2, MFN1, TFAM, COX4i1, and PGC-1α in HCE-2 cells (*n* = 3/group) and the mouse corneal epithelium (*n* = 5/group/treatment). Briefly, control and SKQ1-, PM_10_-, and PM_10_ ± SKQ1-exposed cells were harvested after 24 h and lysed in RIPA buffer with protease and phosphatase inhibitors (Santa Cruz Biotech, Dallas, TX, USA). The resulting supernatants were collected after centrifugation at 12,500 RPM to remove any debris. Protein levels in the supernatant were determined using the Bradford Assay (BioRad, Hercules, CA, USA), as described previously [9]. Samples were diluted with PBS to a concentration of 0.25 μg/μL, according to the manufacturer’s guidelines. Samples were subsequently prepared for analysis on the Abby Simple Western system following the Protein Simple kit and the manufacturer’s guidelines. The primary antibodies SOD2, MFN1, TFAM, COX4i1, and PGC-1α (Proteintech, Rosemont, IL, USA) were used at a 1:50 dilution ratio. The secondary anti-rabbit HRP-conjugated antibody supplied by the manufacturer was used as a detection probe. Subsequently, the samples were re-probed using Abby’s RePlex feature with HRP-conjugated β-tubulin (Proteintech Labs, Rosemont, IL, USA) at a 1:200 dilution. Densiometric readings were obtained using Protein Simple Abby software, Compass for SW’ Version 7.0.0 (Biotechne, Minneapolis, MN, USA), and normalized against β-tubulin. Molecular weights were pre-determined through antibody validation conducted before the experiment. The bands are composites of multiple exposures and are displayed in an HDR format. This fully automated process ensured that the band intensity was neither overexposed nor underexposed. Data were acquired via raw images from the ‘Compass or SW’ program. The entire Abby system is automated, and once the input information is entered, manual changes cannot be made. The program presets all calculations.

### 2.8. Immunofluorescence Staining

For COX4i1 staining, HCE-2 cells were grown on 12 mm coverslips coated with fibronectin, collagen, and albumin (FNC, catalog# NC2000952, Athena ES, Baltimore, MD, USA) in 60 mm Petri dishes; pre-treated with SKQ1; and exposed to PM_10_, as described in the Tissue Culture and Treatments Section. Immunofluorescence was performed as previously described [9]. Cells from the control and SKQ1-, PM_10_-, and PM_10_ + SKQ1-exposed groups (*n* = 3/group) were fixed in 1% paraformaldehyde (PFA) for 10 min at room temperature, permeabilized using 0.2% Triton X-100 for 5 min, blocked with blocking buffer (PBS containing 1% bovine serum albumin (BSA) for 1 h, and incubated with anti-COX4i1 antibody (1:200) diluted in blocking buffer for 1 h. Coverslips were then washed three times, incubated with goat anti-rabbit IgG coupled to CoraLite Plus 488 (1:1500 in blocking buffer) for 1 h, washed, and mounted onto slides using an antifade mounting medium (Vectashield) containing DAPI (Vector Laboratories Inc., Burlingame, CA, USA). A Leica TCS SP8 microscope (Deerfield, IL, USA) was used to image the cells, and all images were processed in a similar manner using Adobe Photoshop version, 7.0.1. All experiments were performed in triplicate.

### 2.9. JC-1 Staining Using Flow Cytometry

HCE-2 cells were grown in 100 mm cell culture dishes and exposed to PM_10_ ± SKQ1, as described above in the Tissue Culture and Treatments Section. The assay was performed following the manufacturer’s instructions. Briefly, the cells were dissociated using TrypLE (ThermoFisher Scientific, Waltham, MA, USA) and transferred to FACS tubes for further analysis. The cells were centrifuged at 300× *g* for 5 min at room temperature. The cells were then resuspended in 100 µL of assay medium. Cells treated with 10 µM carbonyl cyanide m-chlorophenyl hydrazone (CCCP) for 1 h were used as positive controls. A total of 100 µL of JC-1 staining solution was added to each sample individually and incubated for 15 min. JC-1 aggregates were detected in the PE channel, and monomers were detected in the FITC channel. The experiments were performed in triplicate to ensure reproducibility.

### 2.10. JC-1 Live Staining

HCE-2 cells were grown on 12 mm coverslips coated FNC (, Athena ES, Baltimore, MD, USA) in a 12-well plate for 24 h; pre-treated with SKQ1; and exposed to PM_10_, as described in the Tissue Culture and Treatments Section. Cells treated with 10 µM CCCP for 1 h were used as the positive control. Live staining was performed using the JC-1 mitochondrial membrane potential assay kit (Cayman Chemical, Ann Arbor, MI, USA) following the manufacturer’s protocol. Briefly, the cells were incubated with 1 µM JC-1 in cell culture media at 37 °C for 15 min in 5% CO_2_. The cells were washed three times and imaged using a Zeiss Axiophot 200M Apotome microscope (White Plains, NY, USA). All images were processed in a similar manner using Adobe Photoshop, version 7.0.1.

### 2.11. Seahorse Assay

The Seahorse Extracellular Flux Analyzer XFe96 (Agilent, Santa Clara, CA, USA) was used to measure the O_2_ consumption rate in a 96-well format according to the manufacturer’s guidelines. The machine detects changes in O_2_ content in a very small volume of 7 μL above the plated cells using a fluorescence biosensor. Measurements were taken from intact cells at short, repeated intervals, making them noninvasive and physiologically relevant. HCE-2 cells were seeded at 40,000 cells/well in an XFe96 well cell culture microplate previously coated with FNC and exposed to PM_10_ ± SKQ1, as described above in the Tissue Culture and Treatments Section, and incubated for 24 h at 37 °C in 5% CO_2_. A sensor cartridge was placed on the utility plate, and the sensors were submerged in the calibrant in a 37 °C non-CO_2_ incubator overnight. Prior to the assay, the culture medium was removed and replaced with fresh medium, and the cells were placed in a CO_2_-free incubator for 1 h at 37 °C. Stock solutions of oligomycin, FCCP, antimycin A, and rotenone were diluted according to the manufacturer’s instructions and dispensed into Ports A–C of the sensor cartridge. The XF96 analyzer was used to examine basal and maximal respiration, coupling efficiency, ATP production and spare respiration capacity.

### 2.12. Statistical Analysis

Statistical analysis was performed using a one-way ANOVA followed by Bonferroni’s multiple comparison test (GraphPad Prism version 8). The data generated from qRT-PCR, Abby, flow cytometry, and Seahorse were tested for significance set at *p* < 0.05. Tissue culture studies included three biological replicates per group; these studies were repeated once more, while mouse experiments initially used five mice per group; these mouse studies were also repeated once more, resulting in a total of *n* = 10 mice per group. Although a formal power analysis was not performed, the sample sizes were consistent with the ranges typically recommended by power analysis. Randomization and blinding procedures were applied where feasible, particularly in mouse experiments, to minimize bias. The results are presented as the mean ± SEM derived from combined replicates across independent experiments.

## 3. Results

### 3.1. PM_10_ Altered Mitochondrial Function, Which Was Reversed by SKQ1 Pre-Treatment

**Figure 1:**
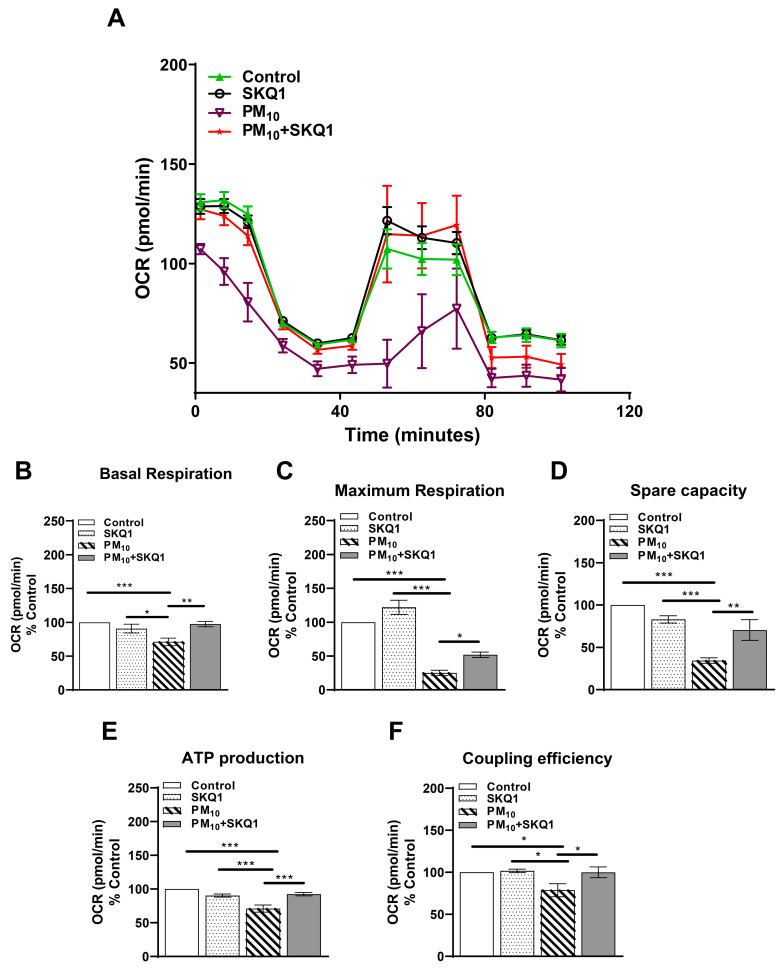
Mitochondrial function in HCE-2 cells following exposure to 100 µg/mL PM_10_ with or without 50 nM SKQ1 was evaluated using a Seahorse bioanalyzer after 24 h. A representative profile of the oxygen consumption rate (OCR) shows a reduced OCR after PM_10_ exposure compared to the control and SKQ1 groups, which was improved by SKQ1 (**A**). PM_10_ significantly reduced basal respiration (**B**), maximum respiration (**C**), spare capacity (**D**), ATP production (**E**), and coupling efficiency (**F**). SKQ1 pre-treatment significantly reversed these effects. Significant changes are denoted by *p*-values indicated by symbols: * *p* < 0.05; ** *p* < 0.01; *** *p* < 0.001. *n* = 3 biological replicates for each group.

To evaluate the effects of PM_10_ on mitochondrial function, we measured mitochondrial respiration by assessing the oxygen consumption rate (OCR) in HCE-2 cells. The data show that exposure to PM_10_ significantly impaired the mitochondrial oxygen consumption rate compared to both the control and SKQ1 groups (Figure 1A). Specifically, after 24 h of PM_10_ exposure, there was a significant reduction in several key parameters of mitochondrial function. Basal respiration (Figure 1B), which reflects the energy demand of the cell under normal conditions, showed a significant decrease after PM_10_ exposure compared to the control (*p* < 0.001) and SKQ1 groups (*p* < 0.05). The maximum respiration (Figure 1C), which indicates the maximal capacity of mitochondria to consume oxygen, showed a significant decline after PM_10_ exposure compared to the control (*p* < 0.001) and SKQ1 (*p* < 0.001) groups. Spare capacity (Figure 1D), which refers to the ability of cells to produce additional ATP through respiration energy demands, showed a significant decrease after PM_10_ exposure compared to the control (*p* < 0.001) and SKQ1 groups (*p* < 0.001). ATP production (Figure 1E), indicating the ability of mitochondria to generate energy, was also significantly reduced after PM_10_ exposure compared to that in the control (*p* < 0.001) and SKQ1 groups (*p* < 0.001). Finally, coupling efficiency (Figure 1F), which measures how effectively oxygen consumption is linked to ATP synthesis, also showed a significant decrease after PM_10_ exposure compared to the control (*p* < 0.05) and SKQ1 (*p* < 0.05) groups. SKQ1 pre-treatment significantly restored the oxygen consumption rate (Figure 1A), basal respiration (Figure 1B, *p* < 0.01), maximum respiration (Figure 1C, *p* < 0.05), spare capacity (Figure 1D, *p* < 0.01), ATP production (Figure 1E, *p* < 0.001), and coupling efficiency (Figure 1F, *p* < 0.05), demonstrating its protective role in preserving mitochondrial function under PM_10_-induced stress.

### 3.2. PM_10_ Reduced the Mitochondrial Membrane Potential, Which Was Reversed by SKQ1 Pre-Treatment

**Figure 2:**
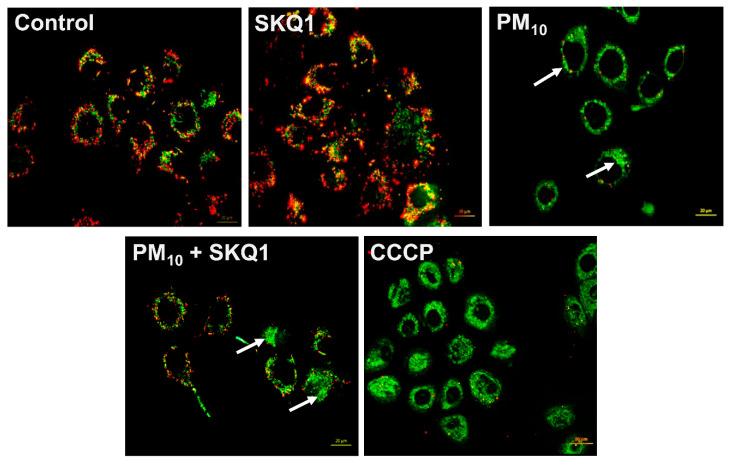
JC-1 staining in HCE-2 cells following exposure to 100 µg/mL PM_10_ in the absence or presence of 50 nM SKQ1. Green fluorescence (monomers) reflects a reduced mitochondrial membrane potential, whereas red fluorescence indicates healthy mitochondria. PM_10_-exposed cells had a lower mitochondrial membrane potential than the control or SKQ1 alone after 24 h. Pre-treatment with SKQ1 before PM_10_ exposure partially restored the mitochondrial membrane potential. CCCP was used as a positive control. Arrows indicate depolarized mitochondria. *n* = 3 biological replicates were used for each group in this study.

**Figure 3:**
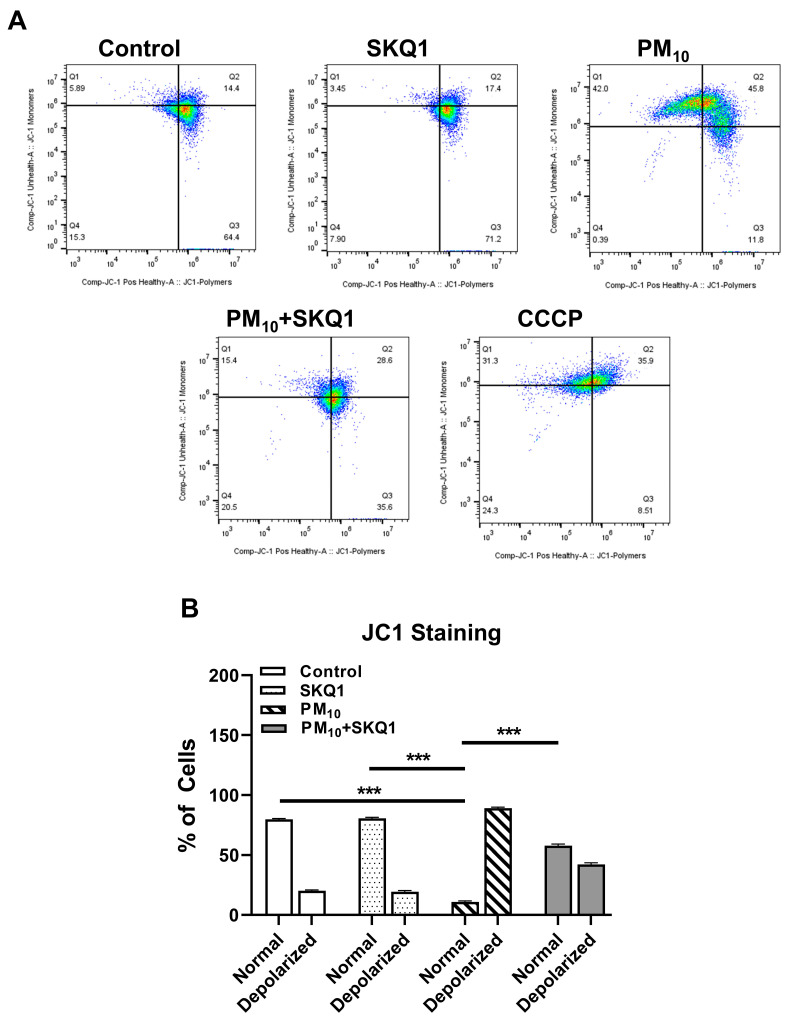
The mitochondrial membrane potential in HCE-2 cells was assessed by flow cytometry using JC-1 dye following exposure to 100 µg/mL PM_10_ with or without 50 nM SKQ1. Scatter plots (**A**) and graphical quantification (**B**) show that PM_10_-exposed cells have more depolarized mitochondria (lower mitochondrial membrane potential) than the control or SKQ1 alone (normal mitochondria) after 24 h. Pre-treatment with SKQ1 before PM_10_ exposure significantly reversed these changes. CCCP was used as a positive control. *** *p* < 0.001; *n* = 3 biological replicates were used for each group in this study.

Following PM_10_ exposure (100 µg/mL), the mitochondrial membrane potential was measured using JC-1 live staining and flow cytometry, as shown in Figure 2 and Figure 3. JC-1 staining showed that cells exposed to PM_10_ predominantly exhibited JC-1 monomers, which emitted green fluorescence (indicated by arrows), and showed a marked absence of JC-1 aggregates, which emitted red fluorescence. This shift from red to green fluorescence indicates a significant reduction in the mitochondrial membrane potential after PM_10_ exposure compared to the control and SKQ1 groups (Figure 2). Flow cytometry scatter plots in Figure 3A also confirm these findings by demonstrating the loss of JC-1 aggregates in PM_10_-exposed cells. A quantitative analysis of the flow data (Figure 3B) further confirmed that PM_10_ significantly increased the number of depolarized mitochondria (*p* < 0.001), along with a significant reduction in the number of mitochondria maintaining a normal mitochondrial potential (*p* < 0.001), relative to the control and SKQ1 groups. SKQ1 pre-treatment significantly reversed these effects, highlighting the protective role of SKQ1 in the mitochondrial membrane’s integrity under PM_10_-mediated stress.

### 3.3. SKQ1 Reversed PM_10_-Induced Effects on Mitochondrial Biogenesis, Quality Control, Structure, and Health

**Figure 4:**
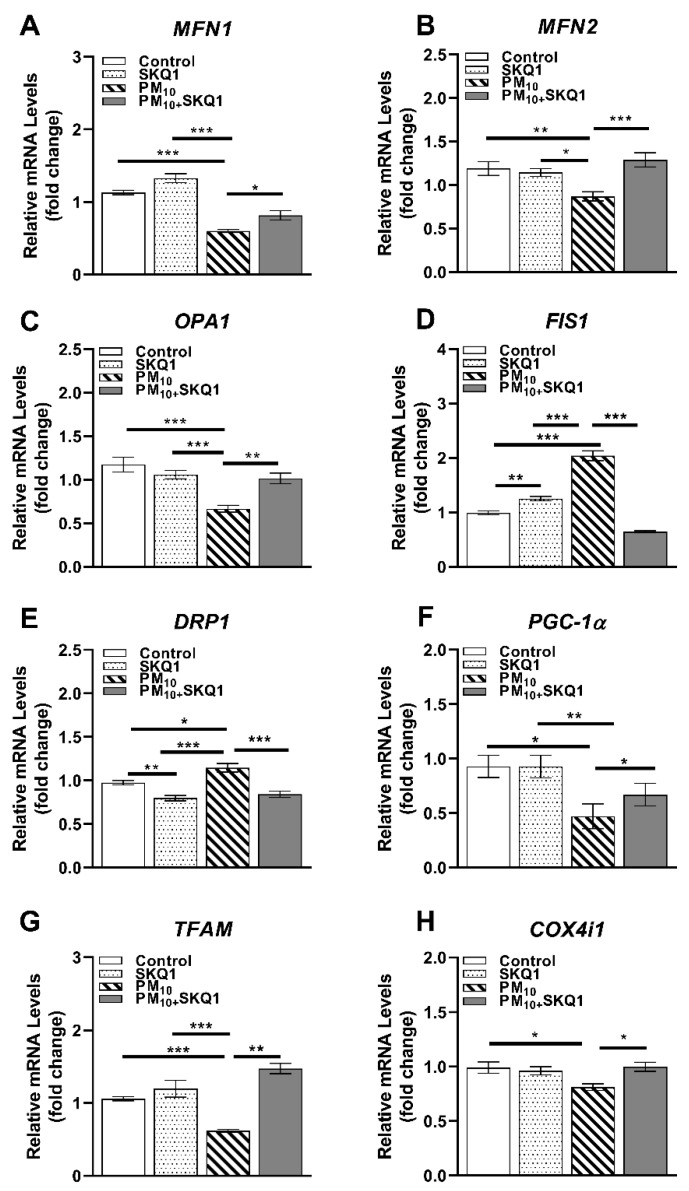
qRT-PCR in HCE-2 cells exposed to 100 µg/mL PM_10_ in the presence or absence of 50 nM SKQ1. Compared to the control and SKQ1 groups, PM_10_ exposure significantly downregulated *MFN1* (**A**), *MFN2* (**B**), *OPA1* (**C**), *PGC-1α* (**F**), *TFAM* (**G**), and *COX4i1* (**H**) but upregulated *FIS1* (**D**) and *DRP1* (**E**) after 24 h. SKQ1 pre-treatment reversed these effects. * *p* < 0.05, ** *p* < 0.01, *** *p* < 0.001, *n* = 3 biological replicates were used for each group in this study.

**Figure 5:**
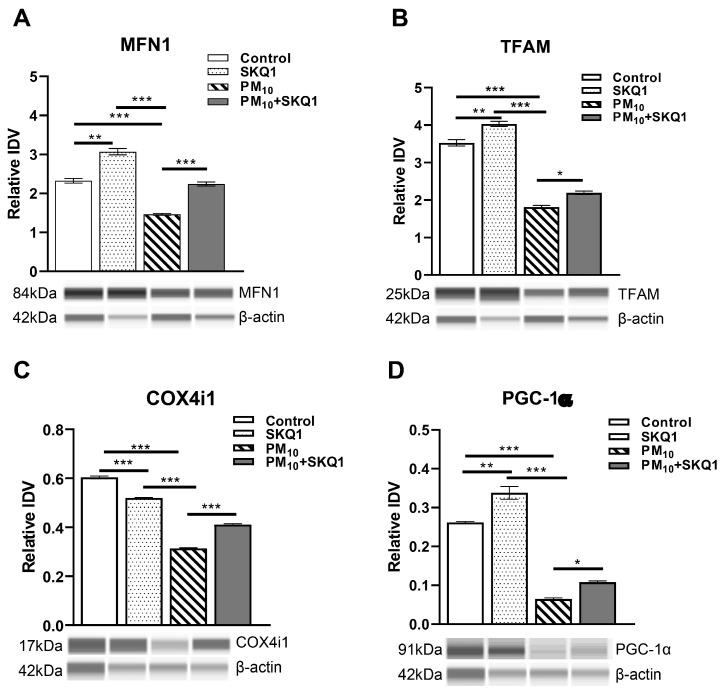
Protein analysis in HCE-2 cells exposed to 100 µg/mL PM_10_ in the presence or absence of 50 nM SKQ1 using the Protein Simple Abby system. PM_10_ exposure significantly downregulated MFN1 (**A**), TFAM (**B**), COX4i1 (**C**), and PGC-1α (**D**) compared to the control and SKQ1 alone after 24 h. Pre-treatment with SKQ1 before PM_10_ exposure reversed these effects. * *p* < 0.05, ** *p* < 0.01, *** *p* < 0.001. *n* = 3 biological replicates were used for each group in this study.

**Figure 6:**
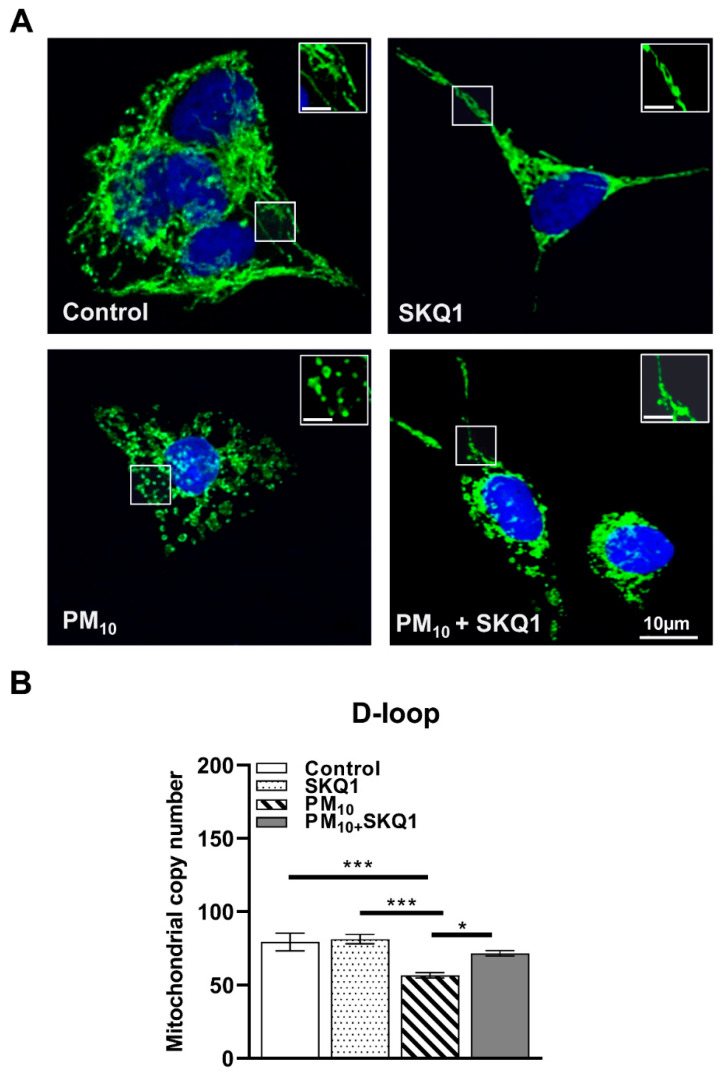
Representative images of mitochondrial morphology (**A**) and DNA copy number (**B**) after exposure of HCE-2 cells to 100 µg/mL PM_10_ ± 50 nM SKQ1. PM_10_ exposure induced mitochondrial fragmentation and reduced mtDNA copy number compared to control and SKQ1 alone after 24 h. SKQ1 pre-treatment partially restored normal elongated morphology and increased reduction in DNA copy number. *n* = 3 biological replicates were used for each group in this study. The white frames indicate enlarged areas to clearly illustrate the differences in morphology. * *p* < 0.05, *** *p* < 0.001.

PM_10_ exposure caused a significant alteration in the expression of key regulators involved in mitochondrial structure, biogenesis, and quality control, as shown in Figure 4, Figure 5 and Figure 6. At the mRNA level (Figure 4), PM_10_ significantly decreased the transcript levels of fusion-related genes *MFN1* (4A, *p* < 0.001, *p* < 0.001), *MFN2* (4B, *p* < 0.01, *p* < 0.05), and *OPA1* (4C, *p* < 0.001, *p* < 0.001) compared to the control and SKQ1 groups, which maintained mitochondrial network integrity through fusion. Additionally, PM_10_ exposure also reduced the expression of genes involved in mitochondrial biogenesis and function, such as *PGC-1α* (4F, *p* < 0.05, *p* < 0.01) and *TFAM* (4G, *p* < 0.001, *p* < 0.001). However, *COX4i1* (4H, *p* < 0.05) levels were only significantly reduced by PM_10_ compared to the control group. These decreases indicate impaired mitochondrial replication, transcription, and respiratory capacity. Conversely, PM_10_ significantly increased the mRNA levels of fission-related genes such as *FIS1* (4D, *p* < 0.001, *p* < 0.001) and *DRP1* (4E, *p* < 0.05, *p* < 0.001) relative to the control and SKQ1 groups. Pre-treatment with SKQ1 significantly increased the transcript levels of *MFN1* (4A, *p* < 0.05), *MFN2* (4B, *p* < 0.001), *OPA1* (4C, *p* < 0.01), *PGC-1α* (4F, *p* < 0.05), *TFAM* (4G, *p* < 0.01), and *COX4i1* (4H, *p* < 0.05) but significantly lowered the levels of *FIS1* (4D, *p* < 0.001) and *DRP1* (4E, *p* < 0.001). Compared to the SKQ1 group, *FIS1* expression (4D, *p* < 0.01) was significantly increased, while *DRP1* expression (4E, *p* < 0.01) was significantly decreased.

At the protein level (Figure 5), PM_10_ exposure similarly caused a significant reduction in MFN1 (5A, *p* < 0.001), TFAM (5B, *p* < 0.001), COX4i1 (5C, *p* < 0.001) and PGC-1α (5D, *p* < 0.001) compared to the control and SKQ1 groups. Pre-treatment with SKQ1 significantly increased the levels of MFN1 (5A, *p* < 0.001), TFAM (5B, *p* < 0.05), COX4i1 (5C, *p* < 0.001) and PGC-1α (5D, *p* < 0.05). Compared to the control group, the SKQ1 group showed significantly higher levels of MFN1 (5A, *p* < 0.001), TFAM (5B, *p* < 0.001) and PGC-1α (5D, *p* < 0.001). In contrast, COX4i1 levels (5C, *p* < 0.001) were significantly reduced in the SKQ1 group compared to the control.

Immunofluorescence analysis (Figure 6A) showed that PM_10_ exposure caused fragmented mitochondria, characterized by small punctate mitochondria instead of the elongated, interconnected network observed in the control and SKQ1 groups. SKQ1 pre-treatment partially restored the elongated mitochondrial structure, suggesting improved mitochondrial dynamics and health. Furthermore, PM_10_-exposed cells showed a significant reduction in the relative mitochondrial DNA (mtDNA) copy number (Figure 6B) measured specifically within the D-loop region, compared to the control (*p* < 0.001) and SKQ1 (*p* < 0.001) groups, suggesting compromised mitochondrial replication. SKQ1 pre-treatment significantly reversed this effect (Figure 6B, *p* < 0.05). These changes collectively suggest that PM_10_ disrupts the balance between mitochondrial fusion and fission, decreases mitochondrial biogenesis, and impairs mitochondrial respiratory components, leading to fragmented and dysfunctional mitochondria. SKQ1 effectively mitigated these effects, indicating the recovery of mitochondrial structure and function.

### 3.4. The Effects of PM_10_ and SKQ1 on the Mouse Corneal Epithelium

**Figure 7:**
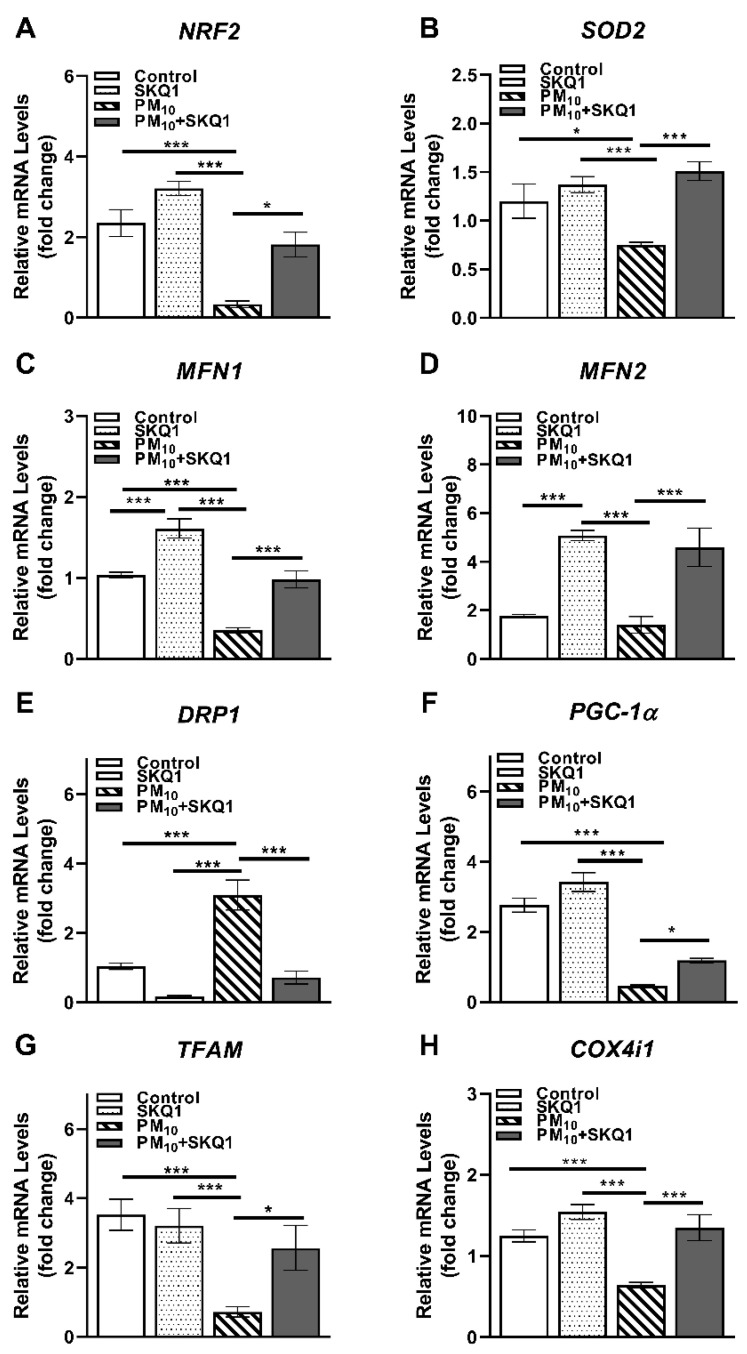
qRT-PCR in mouse corneal epithelium after 2 weeks of exposure to either control air or PM_10_ (0.5–1 mg/m^3^) ± 7.5 µM SKQ1. Data showed that compared to control and SKQ1 groups, PM_10_ exposure significantly downregulated *NRF2* (**A**), *SOD2* (**B**), *MFN1* (**C**), *MFN2* (**D**), *PGC-1α* (**F**), *TFAM* (**G**) and *COX4i1* (**H**) but upregulated *DRP1* (**E**). SKQ1 pre-treatment reduced PM_10_-mediated effects. * *p* < 0.05, *** *p* < 0.001, *n* = 5 biological replicates for each group.

**Figure 8:**
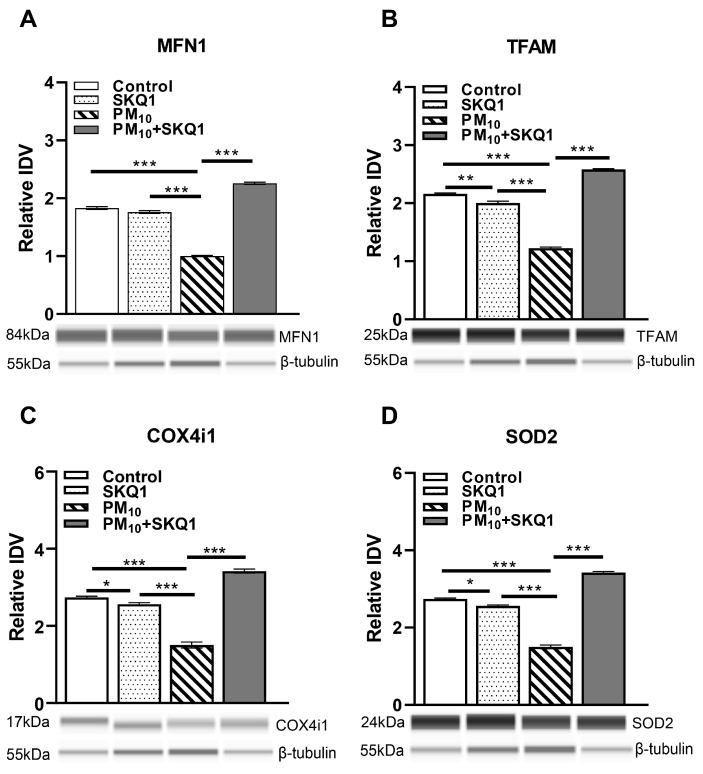
Protein analysis of mouse corneal epithelial sheets after 2 weeks of exposure to either control air or PM_10_ (0.5–1 mg/m^3^) ± 7.5 µM SKQ1 using Protein Simple Abby system. Data indicated that PM_10_ exposure significantly downregulated MFN1 (**A**), TFAM (**B**), COX4i1 (**C**), and SOD2 (**D**) compared to control and SKQ1 alone. Pre-treatment with 7.5 µM SKQ1 reduced PM_10_-mediated effects. * *p* < 0.05, ** *p* < 0.01, *** *p* < 0.001. *n* = 5 biological replicates per group.

The mRNA and protein levels of the regulators of mitochondrial structure, biogenesis, and quality control are shown in Figure 7 and Figure 8. Exposure to PM_10_ caused a significant decrease compared to the control and SKQ1 groups in the mRNA expression of several critical genes: *NRF2* (7A, *p* < 0.001, *p* < 0.001), *SOD2* (7B, *p* < 0.05, *p* < 0.001), *MFN1* (7C, *p* < 0.001, *p* < 0.001), *PGC-1α* (7F, *p* < 0.001, *p* < 0.001), *TFAM* (7G, *p* < 0.001, *p* < 0.001), and *COX4i1* (7H, *p* < 0.001, *p* < 0.001). *MFN2* levels significantly declined compared to SKQ1 (7D, *p* < 0.001) but not compared to the control group. These genes are essential for antioxidant defense, mitochondrial biogenesis, fusion, and electron transport. In contrast, PM_10_ significantly upregulated the mRNA levels of *DRP1* (7E, *p* < 0.001, *p* < 0.001), a key mediator of mitochondrial fission, indicating enhanced mitochondrial fragmentation. Importantly, SKQ1 pre-treatment effectively mitigated (*p* < 0.001) PM_10_-mediated changes in mRNA expression. In addition, compared to the control group, the SKQ1 group showed significantly higher expression levels of *MFN1* (7C, *p* < 0.001) and *MFN2* (7D, *p* < 0.001).

At the protein level (Figure 8), PM_10_ exposure significantly reduced the expression of MFN1 (8A, *p* < 0.001, *p* < 0.001), TFAM (8B, *p* < 0.001, *p* < 0.001), COX4i1 (8C, *p* < 0.001, *p* < 0.001), and SOD2 (8D, *p* < 0.001, *p* < 0.001) compared to the control and SKQ1 groups. SKQ1 pre-treatment significantly counteracted these reductions for MFN1 (5A, *p* < 0.001), TFAM (5B, *p* < 0.001), COX4i1 (5C, *p* < 0.001) and SOD2 (5D, *p* < 0.001). In addition, compared to the control group, the SKQ1 group showed significantly lower expression levels of TFAM (8B, *p* < 0.01), COX4i1 (8C, *p* < 0.05) and SOD2 (7D, *p* < 0.05).

Collectively, these results demonstrate that PM_10_ exposure disrupts mitochondrial homeostasis by reducing biogenesis and fusion markers while upregulating fission markers. SKQ1 pre-treatment is protective and reverses these detrimental changes, thereby preserving mitochondrial integrity and function.

## 4. Discussion

Continuous exposure to the external environment makes the ocular surface highly vulnerable to airborne pollutants, making it an ideal model for studying pollution-mediated changes [41]. Particulate matter (PM) is a major airborne contaminant composed of heterogeneous particles ranging from 0.1 to 10 µm that exert detrimental effects on ocular tissues [42,43]. Based on the aerodynamic diameter, PM is categorized into PM_10_ (coarse particles ≤ 10 µm), PM_2.5_ (fine, ≤2.5 µm), and PM_0.1_ (ultrafine, <0.1 µm) [44]. Epidemiological and clinical studies have linked PM exposure to tear film instability [45], dry eye disease [46], oxidative stress [41] and inflammation [41]. In vitro studies using corneal epithelial cells have shown that PM exposure impairs several cellular processes, including wound healing [47], apoptosis [48,49], autophagy [50], mitochondrial integrity [49], and senescence [9,48]. While the impact of PM_2.5_ on ocular surface toxicity is well documented, relatively few studies have investigated the impact of PM_10_. Previous work from our laboratory established that PM_10_ exposure disrupts redox homeostasis via the dysregulation of the NRF2 pathway both in vitro and in vivo and that the mitochondria-targeted antioxidant SKQ1 confers significant protection against PM_10_ [8,9]. However, the specific effects of PM_10_ on mitochondrial dynamics in corneal epithelial cells remain unclear.

Thus, this study evaluated the impact of PM_10_ on mitochondrial function and structural dynamics in HCE-2 cells (in vitro) and the mouse corneal epithelium (in vivo). Key functional biomarkers, including ROS production, mitochondrial membrane potential, and bioenergetic capacity, reflect oxidative stress and metabolism [51,52,53]. Airborne pollutants, such as PM_2.5_ and PM_10_, are potent inducers of oxidative stress [54], and ROS production is mainly attributed to their heavy metal constituents (cadmium, chromium, copper, iron, and nickel) [55]. Multiple studies have reported that exposure to PM leads to increased mitochondrial ROS production [56,57,58,59]. In our study, we similarly observed elevated mitochondrial superoxide levels in HCE-2 cells using the MitoSOX assay, which detects mitochondrial superoxide, a type of ROS [8]. Mitochondria serve as the main source of intracellular ROS and as prime targets of PM-induced oxidative injury [60,61,62]. Ultrafine particles, PM_2.5_, and PM_10_ have been reported to accumulate within the mitochondrial matrix [63], causing structural damage, including crista disruption and mitochondrial swelling, in tissues such as the lungs, brain, and olfactory epithelium [58,63,64]. These ultrastructural abnormalities are associated with a decline in respiratory capacity [64]. Consistent with these findings in human alveolar basal epithelial cells (A549) [64] and mouse microglial cells (BV2) [65] exposed to PM_2.5_ and other fine particulates, our oxygen consumption rate analyses demonstrated that PM_10_ significantly reduced basal respiration, ATP production, maximum respiratory capacity, and coupling efficiency in HCE-2 cells. Similar mitochondrial respiratory impairments and ATP depletion have been documented in human nasal olfactory mucosal cells [58] and mitochondria isolated from PM_10_-exposed rat lung tissues [66].

Elevated ROS levels can cause the depolarization of the mitochondrial membrane [67]. In this line, we detected the loss of mitochondrial membrane potential in HCE-2 cells treated with PM_10_, corroborating previous reports on lung mitochondria treated with PM_10_ [66]. Mitochondrial morphology, including fission, fusion, and network organization, critically affects mitochondrial function [53]. We found that PM_10_ caused the fragmentation of the normally elongated mitochondrial network in HCE-2 cells, a pattern also described in A549 cells after PM_2.5_ exposure [64]. This observation led us to evaluate the key regulators of mitochondrial dynamics in these cell types. PM_10_ significantly downregulated the fusion proteins MFN1, MFN2, and OPA1 and upregulated the fission mediators DRP1 and FIS1, both in vitro and in vivo. This molecular profile parallels the findings in human vascular endothelial cells (EA.hy926) [68], human bronchial epithelial cells (16HBE) [69], human bronchial epithelial cells (BEAS-2B) [70], and A549 cells [64] exposed to PM_2.5_, where mitochondrial fragmentation is accompanied by suppressed fusion proteins and increased fission.

Mitochondrial size and abundance are correlated with mtDNA copy number, all of which fluctuate according to metabolic demand and extrinsic cellular stressors [71]. The mtDNA copy number serves as a biomarker of mitochondrial integrity [72] and typically decreases under stress. Our data show that PM_10_ significantly reduced the mtDNA copy number in HCE-2 cells, consistent with the reductions reported in pulmonary endothelial cells [73] and A549 cells [64] exposed to PM_2.5_. TFAM, the primary mtDNA packaging protein, is essential for maintaining mtDNA stability [74]. Mitochondrial ROS can suppress TFAM expression and impair biogenesis [74,75]. Our previous data showed that PM_10_ significantly increased mitochondrial ROS production and lipid peroxidation (Malondialdehyde levels) and reduced ATP levels [8], and our current data show decreased PGC-1α, TFAM, and COX4i1 levels, both in vitro and in vivo, after PM_10_ exposure, indicating impaired biogenesis and the disruption of mitochondrial quality control mechanisms. NRF2 regulates the expression of PGC-1α [20], which governs mitochondrial biogenesis [76] and antioxidant defenses, such as SOD2, whose levels were also reduced after PM_10_ exposure. This simultaneous reduction compromises both mitochondrial antioxidant capacity and biogenic regulation, providing a mechanistic explanation for the mitochondrial defects observed following PM_10_ exposure.

The PM_10_ dosage used for in vivo experiments in this study ranged from 0.5 to 1 mg/m^3^. This exposure regimen was selected to mimic the in vivo PM_10_ levels reported in regions such as China, India, and parts of the Middle East [34,35,36]. Similarly, the in vitro PM_10_ dosage was established through dose–response curves [9,39] and was consistently applied in our previous studies [8,9,39], where it effectively induced oxidative stress, barrier dysfunction, and cellular senescence.

The pharmacological modulation of mitochondrial dynamics has been shown to mitigate PM-induced oxidative stress [76], and several natural and synthetic compounds have been investigated for their potential to enhance mitochondrial function [77,78,79]. In this context, we evaluated the mitochondria-targeted antioxidant SKQ1 and demonstrated its efficacy in protecting mitochondrial health from PM_10_-induced toxicity. SKQ1 dosages of 7.5 µM for in vivo experiments and 50 nM for in vitro experiments were determined based on dose–response experiments and are supported by prior published data [8,9,33,39]. The topical application of 7.5 µM SKQ1 demonstrates significant clinical relevance, as it follows the same treatment regimen established in a rabbit model study, which successfully prevented anesthesia-induced dry eye disease [37]. Moreover, ophthalmic SKQ1 solution (Visomitin) has completed phase 3 clinical trials, confirming its efficacy in alleviating the symptoms of dry eye disease, thereby supporting its potential therapeutic use in clinical settings [80]. Collectively, our data indicate that PM_10_-induced ROS are central mediators of mitochondrial dysfunction. The ability of SKQ1 to reverse PM_10_-driven impairments in mitochondrial morphology, respiration, membrane potential, mtDNA stability, and biogenesis strongly supports the notion that oxidative stress is the primary upstream event. The restoration of mitochondrial integrity and function by SKQ1 further demonstrated that the neutralization of mitochondrial ROS can mitigate PM_10_-induced damage.

## 5. Limitations

This study presents valuable and robust data on the effects of PM_10_ on mitochondrial homeostasis on the ocular surface and the protective effects of SKQ1. However, certain limitations of this study should be acknowledged, such as the use of a single immortalized human corneal epithelial cell line and mouse model, as well as a single dose and time point with a relatively small sample size. These factors may influence the extent to which the findings represent the full complexity of the human ocular response to PM_10_ and its broader applicability. The two-week acute exposure model provides important insights but may not fully capture chronic or varying real-world conditions. The protective effects of SKQ1 were tested as a prophylactic agent, at specific doses (50 nM in vitro and 7.5 µM in vivo) and assessed at definite time points, with long-term safety and potential off-target effects to be explored in future studies. Additionally, while immunofluorescence offers valuable information, it has limited resolution and ultrastructural details compared to electron microscopy.

## 6. Conclusions

In conclusion, PM_10_ exposure disrupts multiple aspects of mitochondrial health in corneal epithelial cells, including respiratory function, membrane potential, structural dynamics, mtDNA maintenance, and mitochondrial biogenesis. The protective effects of SKQ1 highlight its therapeutic potential for targeting mitochondrial oxidative stress, thereby preserving ocular surface homeostasis in environments affected by high levels of air pollution.

## Figures and Tables

**Table 1:** Primer sequences used for RT-qPCR (human and mouse).

Human	Sequence	Accession Number
*TFAM*	Forward: 5′-CCAAGAAGCTAAGGGTGATT-3′	*NM_003201.3*
	Reverse: 5′-TGTTTCTTTATTGTGCGACG-3′	
*PGC-1α*	Forward: 5′-TCTGAGTCTGTATGGAGTGACAT-3′	*NM_013261.5*
	Reverse: 5′-CCAAGTCGTTCACATCTAGTTCA-3′	
*DRP1*	Forward: 5′-CTGCCTCAAATCGTCGTAGTG-3′	*NM_012062.5*
	Reverse: 5′-GAGGTCTCCGGGTGACAATTC-3′	
*MFN1*	Forward: 5′-TGGCTAAGAAGGCGATTACTGC-3′	*NM_033540.3*
	Reverse: 5′-TCTCCGAGATAGCACCTCACC-3′	
*MFN2*	Forward: 5′-CTCTCGATGCAACTCTATCGTC-3′	*NM_014874.4*
	Reverse: 5′-TCCTGTACGTGTCTTCAAGGAA-3′	
*FIS1*	Forward: 5′-GTCCAAGAGCACGCAGTTTG-3′	*NM_016068.3*
	Reverse: 5′-ATGCCTTTACGGATGTCATCATT-3′	
*OPA1*	Forward: 5′-TGTGAGGTCTGCCAGTCTTTA-3′	*NM_015560.3*
	Reverse: 5′-TGTCCTTAATTGGGGTCGTTG-3′	
*COX4i1*	Forward: 5′-GAGCAATTTCCACCTCTGC-3′	*NM_001861.6*
	Reverse: 5′-CAGGAGGCCTTCTCCTTCTC-3′	
*18srRNA*	Forward: 5′-CGGCTACCACATCCAAGGAA-3′	*NR_003286.2*
	Reverse: 5′-GCTGGAATTACCGCGGCT-3′	
Mouse	Sequence	Accession Number
*MFN1*	Forward: 5′-ATGGCAGAAACGGTATCTCCA-3′	*NM_024200.5*
	Reverse: 5′-CTCGGATGCTATTCGATCAAGTT-3′	
*MFN2*	Forward: 5′-CAAGTGTCCGCTCCTGAAGG-3′	*NM_133201.3*
	Reverse: 5′-GAACCTCCTTGGCAGACACG-3′	
*DRP1*	Forward: 5′-GGACCCACTAGGTGGCCTTA-3′	*NM_152816.4*
	Reverse: 5′-ACGCTTAATCTGACGTTTGACC-3′	
*PGC-1α*	Forward: 5′-GGTACCCAAGGCAGCCACT-3′	*NM_008904.3*
	Reverse: 5′-GTGTCCTCGGCTGAGCACT-3′	
*TFAM*	Forward: 5′-CACCCAGATGCAAAACTTTCAG-3′	*NM_009360.4*
	Reverse: 5′-CTGCTCTTTATACTTGCTCACAG-3′	
*COX4i1*	Forward: 5′-TCATTGGCTTCACTGCGCTCGT-3′	*NM_009941.3*
	Reverse: 5′-TCCAGCATTCGCTTGGTCTGCA-3′	
*NRF2*	Forward: 5′-TGCCCCTCATCAGGCCCAGT-3′	*NM_010902.5*
	Reverse: 5′-GCT CGG CTG GGA CTC GTG TT-3′	
*β-actin*	Forward: 5′-GATTACTGCTCTGGCTCCTAGC-3′	*NM_007393.5*
	Reverse: 5′-GACTCATCGTACTCCTGCTTGC-3′	

## Data Availability

The original contributions presented in this study are included in the article. Further inquiries can be directed to the corresponding author.
